# Effect of Different Soil Treatments on Production and Chemical Composition of Essential Oils Extracted from *Foeniculum vulgare* Mill., *Origanum vulgare* L. and *Thymus vulgaris* L.

**DOI:** 10.3390/plants12152835

**Published:** 2023-07-31

**Authors:** Antonio Raffo, Filippo Umberto Sapienza, Roberta Astolfi, Gabriele Lombardi, Caterina Fraschetti, Mijat Božović, Marco Artini, Rosanna Papa, Marika Trecca, Simona Fiorentino, Valerio Vecchiarelli, Claudia Papalini, Laura Selan, Rino Ragno

**Affiliations:** 1 CREA-Research Centre for Food and Nutrition, Via Ardeatina, 546, 00178 Rome, Italy; antonio.raffo@crea.gov.it; 2 Rome Center for Molecular Design, Department of Drug Chemistry and Technology, Sapienza University, p.le Aldo Moro 5, 00185 Rome, Italy; filippo.sapienza@uniroma1.it (F.U.S.); astolfi.1766291@studenti.uniroma1.it (R.A.); 3 Department of Environmental Biology, Sapienza University, p.le Aldo Moro 5, 00185 Rome, Italy; gabriele.lombardi@uniroma1.it; 4 Department of Drug Chemistry and Technology, Sapienza University, p.le Aldo Moro 5, 00185 Rome, Italy; caterina.fraschetti@uniroma1.it; 5 Faculty of Natural Science and Mathematics, University of Montenegro, Džordža Vašingtona bb, 81000 Podgorica, Montenegro; mijatboz@ucg.ac.me; 6 Department of Public Health and Infectious Diseases, Sapienza University, p.le Aldo Moro 5, 00185 Rome, Italy; marco.artini@uniroma1.it (M.A.); rosanna.papa@uniroma1.it (R.P.); trecca.1820515@studenti.uniroma1.it (M.T.); laura.selan@uniroma1.it (L.S.); 7 Centro Appenninico del Terminillo “Carlo Jucci”, Perugia University, Via Comunali 43, 02100 Rieti, Italy; simona.fiorentino@unipg.it (S.F.); valerio.vecchiarelli@unipg.it (V.V.); 8 ARSIAL Agenzia Regionale per lo Sviluppo e l’Innovazione dell’Agricoltura del Lazio, Via R. Lanciani 38, 00162 Rome, Italy; c.papalini@arsial.it

**Keywords:** antimicrobial activity, soil fertilization, GC–MS analysis, continuous and fractionated steam distillation

## Abstract

The aim of the study was to investigate how essential oil production and associated chemical composition and related biological activity could be influenced by different cultivation treatments and distillation methods. *Foeniculum vulgare* Mill. (fennel), *Origanum vulgare* L. (oregano) and *Thymus vulgaris* L. (thyme) were cultivated in absence of any fertilizer (control) and in presence of three different fertilizers: a chemical one with augmented mineral phosphorus and potassium, a second added with hydrolyzed organic substance and mineral phosphorus and potassium (organic–mineral) and a third one treated with a high content of organic nitrogen of protein origin (organic). The plants were subjected to steam distillation using two modalities, recycled and continuous, to obtain 32 essential oil samples. Chemical composition analysis was performed using gas chromatography–mass spectrometry; in vitro antimicrobial activity was evaluated using a broth microdilution method. In general, the recycled distillation method appeared to have a slightly higher yield than the continuous method. The “mineral” and “organic–mineral” treatments resulted in a higher yield compared to the “organic” or “control” treatments, and this was particularly evident in the recycled method. The “control” plants had a lower yield of essential oils. Anethole (13.9–59.5%) and estragole (13.4–52.2%) were the main constituents of the fennel oils; *p*-cymene and its derivatives carvacrol and thymol were the main constituents of the oregano and thyme samples. The antimicrobial activity of the thyme oils on *Staphylococcus aureus* ranged from 0.31 to 0.16% (*v/v*); a lower effect of the oregano samples and no activity of the fennel samples were observed. The essential oils failed to inhibit the growth of *Pseudomonas aeruginosa* strains.

## 1. Introduction

Plants and their extracts contain phytochemical bioactive compounds that are commonly used as antimicrobial and antibiofilm agents [1]. Novel antibacterial targets and compounds derived from natural plants will help to develop innovative antimicrobial strategies and improve existing ones. Mixtures of volatile organic compounds, known as essential oils (EOs), can be extracted from many plants. EOs are aromatic and oily liquids that can be extracted from virtually any plant using various methods, of which steam distillation is the most commonly used for their commercial production [2]. 

The origins of EO production date back thousands of years, and they have been used for medicinal purposes for at least as long [3]. Today, EOs are mainly used for aromatherapy, skin care and alternative healing practices, and only a few applications have been reported for medical purposes.

The chemical composition of EOs and thus their physical and biological properties are strongly influenced by several factors. Some of these factors are related to either the way the plants are treated during their growth [4] or the time of harvest [5], while others are instrumental factors such as the extraction method [6] and/or the duration of extraction [7,8,9,10,11,12]. 

Irrespective of the treatment of the plant material or the way EOs are produced from it, the scientific world is witnessing a global EO market that is predicted to grow at a compound annual growth rate (CAGR) of about 8–10% in the next few decades [13]. The global EO market size was estimated at USD 18.6 billion in 2020 and is expected to be driven by increasing demand from major end-use industries such as food and beverages, personal care and cosmetics and aromatherapy. Unlike conventional drugs and pharmaceuticals, EOs have few side effects at the suggested dosage [14], including allergic reactions and phototoxic effects, and only a few EOs exhibit necrotic, narcotic, nephrotoxic, hepatotoxic or carcinogenic effects. Nevertheless, most side effects are caused by their misuse [15,16], due in general to a wrong dosage and automedication.

The main drawback of the use of EOs is either their low compositional stability [17] or the difficulty of producing EOs with a constant composition even from the same plant material [18]. Nevertheless, the encapsulation of EOs or other methods for their vehiculation are constantly under investigation [19,20], and along these lines, applications of machine learning algorithms aimed at rational design of EO mixtures represent an alternative for indirectly standardizing EOs in a dynamic way [21,22,23,24,25,26].

EOs have been traditionally used for hundreds of years as natural medicines to combat pathogens, including bacteria, fungi and viruses [27]. Previous studies have focused on the use of plant extracts as alternative treatments for infectious diseases. Among the most studied EOs are those obtained from cinnamon, thyme, mint species, oregano, fennel and marjoram [28]. 

The antimicrobial activity of several EOs is often associated with damage to the cell wall and membranes, leading to cell lysis with leakage of cell contents [2]; nevertheless, although not in the antimicrobial field, biochemical molecular biology studies are starting to elucidate some other mechanisms [29,30]. In addition, scientific evidence shows that EOs effectively kill bacteria without promoting the acquisition of resistance [31]. In fact, bacteria do not develop resistance to multi-component drugs such as EOs because of their multi-target action. 

As part of an ongoing project to investigate how EO production and the associated chemical composition and bioactivity can be influenced by different cultivation treatments, three well-known aromatic plants were harvested and subjected to EO distillation: *Foeniculum vulgare* Mill. (FV, fennel), belonging to the *Apiaceae* family, and two species of *Lamiaceae*, *Origanum vulgare* L. (OV, oregano) and *Thymus vulgaris* L. (TV, thyme). The plants were grown under different soil treatments and then harvested and subjected to EO distillation. For the distillation, classical steam distillation (SD) was performed through a Clevenger-type apparatus [32]. SD was performed on different harvested plant samples to collect the condensed EOs/water vapors continuously (CD) or in a recycled manner (RD). The chromatography–mass spectrometry (GC–MS) analysis of the residue result showed *p*-cymene, thymol and carvacrol as the main constituents of the EOs from OV and TV, while the EOs from FV contained a predominance of estragole and anethole. To complete their characterization, 32 EO samples were then tested for their antimicrobial ability against four different bacterial strains belonging to either Gram-positive *Staphylococcus aureus* or Gram-negative *Pseudomonas aeruginosa* species.

## 2. Results

### 2.1. EO Extraction

In total, 32 EOs samples were obtained: 13 from FV, 11 from OV and 11 from TV. The yields of essential oils ranged from 0.011 to 0.098%. The FV and OV EOs showed a higher yield (Table 1).

#### 2.1.1. EOs from Fennel

The EOs extracted from the FV plants showed different yields as a result of either different soil treatments or distillation methods. In general, RD distillation produced a higher percentage of EO, while all treatments led to an increased percentage of EO compared to the control, with a maximum reached for the organic–mineral treatment (0.50% for RD distillation). The amount of dried plant available in some cases enabled performing the distillation in duplicate, as in the case of the control with the CD method (FV01 and FV02), the mineral-treated plants extracted with the CD method (FV04 and FV05) and the organic-treated plants and extracted with the RD method (FV08 and FV09), or in triplicate, as in the case of the organic–mineral extracted with the RD method (FV11, FV12 and FV13).

#### 2.1.2. EOs from Oregano

Similar to FV, the RD distillation of OV provided a higher amount of EO for both control and treated crops, while the treatment that provided the highest amount of EO was the mineral one (0.45–0.65% of EO for the RD distillation). Similarly to the FV extraction, the mineral- and organic–mineral-treated plants were extracted in duplicate with the RD method, while the organic-treated plants were extracted in duplicate with the CD method. 

#### 2.1.3. EOs from Thyme

As for the other two plants, the RD distillation method resulted in higher yields of EO. However, each treatment yielded a higher amount of EO, the organic–mineral being the one that yielded the highest percentage of EO using the RD distillation method (0.57% of EO for the RD distillation). For the mineral-treated plants, duplicate extractions were performed with the CD method, while for the organic-treated plants, duplicate extractions were performed with either the CD or RD method.

### 2.2. EO Chemical Analysis

The compositions of the EOs were analyzed using gas chromatography (GC) coupled with mass spectrometry (MS), which was aimed at the identification and relative quantification of individual components within each sample. 

#### 2.2.1. EOs from Fennel

GC–MS analysis of the FV EOs revealed a total of 34 chemical constituents (FV01–FV13, Table 2 and Table 3). They accounted for more than 99% of the total EO content. A total of 28 compounds were identified belonging to the classes of monoterpene hydrocarbons (9), oxygenated monoterpenes (12), sesquiterpenes (2) and phenylpropanoids (5). The phenylpropanoids anethole (from 13.9 to 59.5%) and estragole (from 13.4 to 52.2%) were the main constituents of the FV EOs: their sum was relatively stable across all EOs, ranging from 57.54% (FV09) to 79.35% (FV11). The two phenylpropanoids were followed by limonene, *p*-cymene and α-pinene as the main monoterpene hydrocarbons and fenchone as the main oxygenated monoterpene. Four different chemical profiles were observed when evaluating the total EO composition: a first profile (FV04, FV05, FV08, FV09, FV10, FV11 and FV13), characterized by intermediate levels of anethole and estragole; a second profile (FV02, FV08 and FV12), with high levels of estragole; a third profile (FV03 and FV07), characterized by high levels of anethole and limonene and a fourth profile (FV07) high in anethole and low in estragole.

#### 2.2.2. EOs from Oregano 

The GC–MS analysis of the OV EOs (OV2–OV11, Table 4 and Table 5) enabled the detection of 43 chemical constituents, which explained from 91% to more than 99% of the total composition; 13 identified compounds belonged to the monoterpene hydrocarbon class, 14 were oxygenated monoterpenes, 12 were sesquiterpenes and 4 could be classified as other compounds. Additionally, 2 cymyl compounds, carvacrol and thymol, were the main constituents in all EOs considered: in most EO samples, their sum amounted to more than 80% of the total constituents. Lower levels of carvacrol and thymol were observed in OV05, OV08 and OV11 with percentages of 43.9%, 58.9% and 46.5%, respectively. 

Among the two direct precursors of the above-mentioned cymyl compounds, γ-terpinene (less than 6.2%) and *p*-cymene (from 1.5 to 36.5%), the latter was present at a significantly higher level. Nine EOs (OV02, OV03, OV04, OV06, OV07, OV08, OV09, OV10 and OV11) could be clearly assigned to the carvacrol chemotype, with carvacrol content ranging from 44.7 to 81.4%. Only OV05 seemed to belong to the thymol chemotype with a thymol concentration of 40.9%. Three different profiles could be recognized on the basis of the chemical compositions in the considered set of OV EOs: a first profile with high levels of carvacrol and low levels of thymol and *p*-cymene; a second profile, represented by OV11, with intermediate levels of carvacrol and *p*-cymene and very low levels of thymol and a third profile, represented by OV05, with high levels of thymol, intermediate levels of *p*-cymene and very low levels of carvacrol. It was not possible to analyze the EOs extracted using CD from the control plant.

#### 2.2.3. EOs from Thyme 

In the nine TV EO samples analyzed (TV03–TV11, Table 6 and Table 7), forty-four compounds were detected, representing 88–97% of the total EO content. The identified compounds belonged to the following chemical classes: monoterpene hydrocarbons (10), oxygenated monoterpenes (16), sesquiterpenes (12), phenylpropanoids (2) and other (4). Among the cymyl compounds, thymol and carvacrol, the former was present as the major EO constituent in all thyme EOs, ranging from 35.7 to 64.7%, whereas carvacrol comprised below 10% in all samples. Interestingly, the *p*-cymene content was higher than that of carvacrol in four EOs—TV05, TV07, TV09 and TV11—ranging from 19.0 to 32.2%. In these EOs, a relatively high level of *p*-cymene corresponded to a relatively low level of thymol. Three profiles of thyme EOs could be identified on the basis of the chemical compositions: a first profile (TV03, TV04, TV06, TV08 and TV10) with high levels of thymol and low levels of *p*-cymene; a second profile (TV09 and TV11) with intermediate levels of thymol and *p*-cymene and a third profile (TV05 and TV07) with relatively high levels of *p*-cymene and relatively low levels of the main compound thymol. It was not possible to analyze the EOs of the control plants.

### 2.3. EO Antimicrobial Activity Evaluation

In vitro antimicrobial activity of the EOs was evaluated on *S. aureus* and *P. aeruginosa* reference strains using broth microdilution methods. An appropriate dilution (as reported by the National Committee for Clinical Laboratory Standards NCCLS, 2023) of 10^6^ cfu/mL of each bacterial culture in the exponential phase was used (Table 8, Table 9 and Table 10).

No antimicrobial activity was observed against *S. aureus* strains by any of the EOs extracted from FV (Table 8). 

A lower antimicrobial effect of the EOs derived from OV was observed, ranging from 0.16 to 2.5% (*v*/*v*), except for OV05, for which no antimicrobial activity was observed on *S. aureus* ATCC 25923. The antimicrobial activity of the TV EOs on *S. aureus* ranged from 0.31 to 0.16% (*v*/*v*). 

In particular, five samples (OV04, OV07, OV08, TV06 and TV11) were actually able to inhibit the growth of either ATCC 6538P or ATCC 25923 *S. aureus* strains at MIC values as low as 0.16% *v*/*v*, while OV03, OV07, TV06, TV07, TV08 and TV11 showed MIC values of 0.31% *v*/*v* (Table 9 and Table 10). All samples tested against either *P. aeruginosa* PAO1 or PA14 strains were unable to inhibit bacteria grown at the higher EO concentration used (5% *v*/*v*).

## 3. Discussion

A series of aromatic plants were cultivated with different soil treatments in order to study the effect of fertilization variation on EO production and EOs’ chemical and biological profiles. The yield percentages varied depending on the extraction method and soil treatment (Table 1). In general, the RD method seems to have slightly higher yield percentages compared to the CD method (except for OV2 and OV4, which gave higher yield percentages with CD). 

It is important to note that the “mineral” and “organic–mineral” treatments resulted in higher EO yield percentages compared to the “organic” or “control” treatments. This is particularly evident in the RD method, where the organic–mineral treatment gave high yield percentages (0.636% for OV04 and 0.499% for FV11). Moreover, it is noticeable that the “control” plants, which were not treated with any specific fertilization method, generally had a lower EO yield compared to the other treatments. This is particularly evident for the control plants which yielded percentages of 0.152% (OV02), 0.130% (FV03) and 0.104% (FV02).

GC–MS analyses of all samples showed quantitative variability in the EO composition and relative concentration, which varied considerably depending on the soil treatment. 

Regarding the fennel extracts FV01–FV13, the amount of the main components, namely, anethole and estragole, varied between 25.50% and 60.00%. A similar profile has been reported indicating that phenylpropenes estragole and anethole are the major constituents of EOs extracted from FV aerial parts, which changed during plant development [33]. Some of the compounds present in significant amounts include α-pinene, β-pinene, β-myrcene, α-phellandrene, *p*-cymene, limonene, fenchone, estragole, anethole, carvacrol and 4-methoxycinnamaldehyde.

Regarding the possible influence of the soil treatment, although important percentage variability could be observed from the chemical analysis of the EOs, somehow the treatment seemed to influence the chemical profile of the FV EOs. In particular, anethole was the most abundant component in all the analyzed controls (FV01, FV02 and FV03), depending on both the extraction method and the treatment, and its percentage was increased to almost 60% (FV9) when treated with organic fertilizer and extracted with the CD method. In all other cases, the percentage of anethole was always lower than in the controls. Differently, in the case of estragole, in general, all treatments maintained the percentage of the controls with a definitive increase for extraction with the RD method in all treatments (FV06, FV08 and FV12). No correlation can be made for the antimicrobial activity and the treatments because the FV EOs were not active at the higher concentration used.

For the OV EOs, a total of 43 compounds were identified, and the main constituents were carvacrol (up to 60%), thymol (between 4% and 21%) and *p*-cymene (between 4% and 36%). These data are consistent with those found in the literature and listed in the freely accessible EO database currently under development (the eo.3d-qsar.com (accessed on 15 July 2023)), and they align with those found in *Origanum vulgare* genotypes recently reported [34]. From a survey, the main chemical components of OV EOs are reported to be carvacrol (55–81%) and thymol (3–40%), with some important levels of α-terpinene, *p*-cymene and linalool. The different soil treatments compared to the control seem to affect mainly the *p*-cymene content. In particular, the percentage of *p*-cymene in most of the EOs increased from 2.5 (OV10) to 22 times (OV11), while such a large variation was not observed for the other components.

The OV EOs showed different antimicrobial activity on *S. aureus* reference strains depending on the treatments used for plant cultivation and, correspondingly, on the different composition of each EO. In particular, the main component carvacrol seemed to be associated with better antimicrobial activity, having a concentration higher than 50% in OV3, OV4, OV5, OV6 and OV8 (54–78%) compared to OV9 and OV10 (3–44%). Conversely, *p*-cymene was more abundant in OV9 and OV10 (23–36%) and seemed to have a negative effect on antimicrobial potency. Spathulenol, although in very low concentrations (0.085–0.344%), was found only in the OV samples that showed antimicrobial activity. The latter finding is consistent with the concept that the antimicrobial activity of a complex mixture such as an EO is also due to compounds present at very low concentrations and not only to the more abundant ones. Unfortunately, it was not possible to determine the MIC for the control because of the low amount available. Nevertheless, it seemed that the mineral and organic soil treatments yielded slightly more potent EO compositions than those obtained with the organic–mineral treatment. 

Analyses show that the thyme EOs samples (TV03–TV11) contained 44 recognized compounds, with *p*-cymene (present from about 2% to 32%) and thymol (about 35–64%) as the main components, suggesting that the EOs belong to the thymol chemotype. The other components were present in a total amount of less than 15% [35]. As reported for the OV EOs, these data are in good agreement with the literature which indicates that the main chemical components of thyme EO are thymol (20–60%) and carvacrol (5–20%) as well as *p*-cymene, α-terpinene and linalool (data from eo.3d-qsar.com (accessed on 15 July 2023)). Because of the lack of both chemical composition and microbiological data on the EOs extracted from the control plants, it was not possible to verify any influence of the soil treatment. Nevertheless, the thymol content of the extracts had some fluctuation, giving higher percentages with the CD extraction method for all three treatments (TV04, TV06 and TV10). On the other hand, while the thymol percentages were lower (TV05, TV07 and TV09), the *p*-cymene concentration was higher (TV05, TV07 and TV09), but this content variability could not be correlated with either the extraction method or the soil treatment.

## 4. Materials and Methods

### 4.1. Plant Material and Soil Treatment 

FV, OV and TV plants were grown at the Stazione di Base del Centro Appenninico del Terminillo “Carlo Jucci” in Rieti (Italy). The first transplanting was completed in September 2016. The plants were planted in twelve separate experimental square plots (four on each of the three rows) and were treated differently to perform four separate experiments (Table 11). The plants were harvested in the summer of 2018; the plant material was then dried for 21 days in an aerated, shaded area; sealed and stored in a cabinet until further analysis.

### 4.2. EO Steam Distillation 

The dried aerial parts of FV, OV or TV plants were subjected to steam distillation, collecting the condensate for a period of 1 h. Steam distillation was carried out in two modalities, (1) recycled distillation (RD), from which the water/oil double phase was allowed to accumulate without interruption, and (2) continuous distillation (CD) [7,8,9,10,11,36,37,38], the conventional form of EO distillation, where the condensed water/oil layers were collected directly in a bottle during distillation. The distillation time was arbitrarily set at 1 h, which is also a more productive duration [8,9]. 

For distillation, the plant material was placed in the upper part of a chamber of a Clevenger-type steel apparatus, and the steam generated by the boiling water in the lower part passed through the plant material, softening its cells and allowing the EO to escape in vaporized form. Once released, tiny droplets of EO formed and mixed with the steam and converged into a cooling system. All EOs produced had a lower specific gravity than water, formed a layer on the condensed water, and were easily separated with a separating funnel [8,39]. The separated EOs were extracted twice with diethyl ether (Sigma-Aldrich, Milan, Italy), and the collected EO/diethyl ether phases were dried over anhydrous sodium sulfate (Sigma-Aldrich, Italy). The solvent was evaporated to yield the dried EOs, which were stored in brown glass vials at −18 °C in the dark until further analysis. 

### 4.3. EO Chemical Analysis 

EOs were diluted in methanol (1:20 *v*/*v*) prior to GC analysis. GC analyses were performed on an Agilent 6890 5973 N GC–MS system equipped with a quadrupole mass filter for mass spectrometric detection (Agilent Technologies, Palo Alto, CA, USA) and a DB1-MS column (0.25 mm × 60 m, 0.5 μm film thickness; J&W, Agilent Technologies, Palo Alto, CA, USA) for GC separation. The chromatographic conditions were as follows: 1 µL volume, split injection (50:1 ratio), injector temperature at 250 °C, oven temperature program from 60 °C (1 min) to 200 °C at 4 °C min^−1^ and then to 280 °C (5 min) at 50 °C min^−1^, constant He carrier gas flow was 1.5 mL min^−1^, corresponding to a linear velocity of 32 cm s−1. The MS detector was operated in electronic impact ionization mode at 70 eV; transfer line, source and quadrupole temperatures were set at 300, 230 and 150 °C, respectively. Detection was performed in full-scan mode over the 33−300 amu mass range. Identification of chemical compounds was performed by comparison of linear retention indices (LRIs) and mass spectra of chromatographic peaks with those obtained on standard solution of pure reference compounds (purchased from Merck, Sigma-Aldrich, Milan, Italy). Linear retention indices (LRIs) were determined by analyzing a standard solution of C7–C30 saturated alkanes under the same conditions as for the EOs and by applying the equation proposed by van Den Dool and Kratz [40]. When a pure compound was not available, the tentative identification was based on the comparison of the determined LRIs with those reported in the literature [41] and in the NIST Chemistry WebBook database (NIST, 2021) and on the comparison of the mass spectra with those reported in the NIST/EPA/NIH Mass Spectra Library 2005 (Supplementary Material Tables S1–S3). Information on composition of EOs was reported as the relative GC–MS % abundance of all detected compounds, which was calculated on the basis of peak areas in the GC Total Ion Current profile detected using the full-scan mode. Each EO sample was analyzed in duplicate. All the quantifications were conducted in agreement with the indication reported by Cachet et al. [42].

### 4.4. Bacterial Strains and Culture Conditions

The following reference strains were used in this study: *S. aureus* ATCC 6538P (6538P) and *S. aureus* ATCC 25923 (25923), conventionally used for antimicrobial testing, and *P. aeruginosa* ATCC PAO1 (PAO1) and *P. aeruginosa* ATCC PA14 (PA14), recognized as moderately and highly virulent, respectively [43]. Bacterial strains were stored in frozen glycerol stocks, plated on fresh Brain Heart Infusion agar plates (BHI, Oxoid, Basingstoke, UK) and incubated at 37 °C for 18 h. They were then subcultured under vigorous agitation (180 rpm) in BHI broth to provide fresh cultures.

### 4.5. Determination of Minimal Inhibitory Concentration (MIC)

MIC was determined according to the guidelines of the Clinical Laboratory Standards Institute (CLSI, 2023). Mother stock solutions were prepared by solubilizing each EO in DMSO to a final concentration of 50% (*v*/*v*). A series of solutions were prepared from each EO mother stock with twofold serial dilution. A total of 8 concentrations were used in the range of 5–0.037% (*v*/*v*). The experiments were performed in quadruplicate. The MIC was determined as the lowest concentration at which observed bacterial growth was inhibited. 

## 5. Conclusions

Here, a first pioneering investigation of the variability in EO chemical composition influenced by either different soil treatments and/or distillation methods is reported. At first glance, the EO composition seems to be altered depending on both the distillation method and soil treatment. To some extent, the variability in chemical composition also influenced the microbiological effect in inhibiting *S. aureus* viability. More data are being collected with the goal of applying machine learning algorithms to shed some light on the difficulty of standardizing EO behavior through established cultivation and extraction protocols.

## Figures and Tables

**Table 1:** EO extraction yields listed below represent the percent of EO obtained per weight of plant material.

EO Name	Soil Treatment	Distillation Method	Yield (%)
FV01	control	CD ^1^	0.13
FV02	control	CD	0.10
FV03	control	RD ^2^	0.19
FV04	mineral	CD	0.18
FV05	mineral	CD	0.16
FV06	mineral	RD	0.21
FV07	organic	CD	0.18
FV08	organic	RD	0.21
FV09	organic	RD	0.23
FV10	organic–mineral	CD	0.16
FV11	organic–mineral	RD	0.50
FV12	organic–mineral	RD	0.30
FV13	organic–mineral	RD	0.25
OV01	control	CD	0.10
OV02	control	RD	0.15
OV03	mineral	CD	0.19
OV04	mineral	RD	0.64
OV05	mineral	RD	0.45
OV06	organic	CD	0.21
OV07	organic	CD	0.23
OV08	organic	RD	0.26
OV09	organic–mineral	CD	0.16
OV10	organic–mineral	RD	0.18
OV11	organic–mineral	RD	0.20
TV01	control	RD	0.15
TV02	control	CD	0.19
TV03	mineral	CD	0.25
TV04	mineral	CD	0.25
TV05	mineral	RD	0.27
TV06	organic	CD	0.18
TV07	organic	CD	0.23
TV08	organic	RD	0.28
TV09	organic	RD	0.25
TV10	organic–mineral	CD	0.21
TM11	organic–mineral	RD	0.57

^1^ Continued Distillation; ^2^ Recycled Distillation.

**Table 2:** Chemical composition of fennel EOs FV01–FV07. Data are expressed as relative GC–MS% abundance of all detected components.

EO Component	RI ^1^	FV01	FV02	FV03	FV04	FV05	FV06	FV07
α-pinene	933	0.73	2.39	2.60	1.57	4.49	3.57	1.66
sabinene	968	0.07	0.08	0.11	0.08	0.11	0.23	0.09
β-pinene	974	0.29	0.22	0.25	0.12	0.47	0.29	0.15
β-myrcene	982	0.32	0.57	0.67	0.37	0.76	0.69	0.47
α-phellandrene	1000	0.11	4.17	2.11	0.18	0.21	3.12	0.36
3-carene	1008	1.52						
*p*-cymene	1014	6.11	2.73	3.57	3.18	2.29	4.89	0.87
limonene	1023		8.95	15.74	9.04	16.83	11.43	15.58
γ-terpinene	1050		0.22				0.26	0.06
fenchone	1071	6.31	2.01	1.66	6.36	2.20	4.33	4.33
linalool	1084		0.11					
fenchylalcohol	1105		0.13	0.20		0.16		
*cis*-*p*-menth-2,8-dienol	1118					0.08		
camphor	1124	0.11	0.06		0.11			0.06
4-terpineol	1165	0.05	0.15			0.06		
estragole	1180	32.86	36.16	15.56	37.75	35.77	52.21	13.44
verbenone	1185	0.06	0.19	0.23	0.08	0.13	0.28	
fenchylacetate, endo	1209	0.35	0.34	0.62	0.33	0.63	0.66	0.34
p-anisaldehyde	1215	2.70	0.15	1.05	2.98	1.88	0.70	0.91
fenchylacetate, exo	1224	1.84	2.41	4.31	1.27	3.12	1.97	1.34
anethole	1264	45.34	36.36	49.88	35.23	29.87	13.92	59.51
isobornyl acetate	1272	0.08	0.07	0.13		0.08	0.10	0.08
carvacrol	1282	0.22	1.43	0.24	0.12	0.24		0.33
2,3-dimethylhydroquinone	1333	0.12	0.32			0.10		0.30
anisyl methyl ketone	1343	0.07			0.13			
β-caryophyllene	1423	0.06	0.40	0.23				0.08
4-methoxycinnamaldehyde	1520	0.11			0.16	0.14	0.06	
caryophyllene oxide	1576		0.07	0.12				
Total		99.43	99.69	99.28	99.06	99.62	98.71	99.96

^1^ Retention indexes relative to standard mixture of n-alkanes on DB1-MS column.

**Table 3:** Chemical composition of fennel EOs FV08–FV13. Data are expressed as relative GC–MS% abundance of all detected components.

EO Component	RI ^1^	FV08	FV09	FV10	FV11	FV12	FV13
α-pinene	933	5.52	3.20	3.74	0.20	2.79	5.56
sabinene	968	0.18	0.10	0.12		0.14	0.17
β-pinene	974	0.50	0.24	0.41		0.23	0.49
β-myrcene	982	0.87	0.42	0.69	0.12	0.44	0.83
α-phellandrene	1000	5.42		0.46	0.14	0.34	1.51
3-carene	1008						
*p*-cymene	1014	5.63	3.39	3.73	1.99	4.43	6.83
limonene	1023	10.75	16.58	15.87	2.67	11.27	13.92
γ-terpinene	1050	0.13			0.06		
fenchone	1071	1.76	5.50	1.73	4.92	4.28	2.06
linalool	1084						
fenchylalcohol	1105	0.14		0.15			0.09
*cis*-*p*-menth-2,8-dienol	1118		0.12	0.09			
camphor	1124				0.09	0.07	
4-terpineol	1165			0.06	0.06		
estragole	1180	48.34	30.62	25.86	37.04	49.04	38.84
verbenone	1185	0.28		0.23	0.12	0.13	0.20
fenchylacetate, endo	1209	0.86	0.77	0.69	0.68	0.43	0.32
p-anisaldehyde	1215	0.56	7.09	2.11	3.20	1.59	1.83
fenchylacetate, exo	1224	3.74	2.59	2.92	3.18	2.83	1.88
anethole	1264	14.38	26.92	39.10	42.31	20.32	24.08
isobornyl acetate	1272	0.11	0.09	0.11	0.14	0.10	
carvacrol	1282			0.30	0.65		0.10
2,3-dimethylhydroquinone	1333		0.14	0.14	0.07		
anisyl methyl ketone	1343		0.43	0.08	0.21	0.16	
β-caryophyllene	1423	0.07		0.09			
4-methoxycinnamaldehyde	1520		0.28	0.13	0.23	0.19	0.11
caryophyllene oxide	1576				0.07		
Total		99.24	98.48	98.81	98.15	98.78	98.82

^1^ Retention indexes relative to standard mixture of n-alkanes on DB1-MS column.

**Table 4:** Chemical composition of oregano EOs OV02–OV06. Data are expressed as relative GC–MS% abundance of all detected components.

EO Component	RI ^1^	OV02	OV03	OV04	OV05	OV06
α-thujene	925		0.07	0.20	0.71	
α-pinene	933		0.08	0.14	0.48	
β-thujene	937					
camphene	947		0.04	0.06	0.28	
1-octen-3-ol	961	0.74	0.91	0.89	0.63	0.37
3-octanone	964	0.17	0.20	0.22	0.05	0.08
sabinene	968					
β-pinene	974		0.04	0.07	0.15	
3-octanol	978		0.04		0.07	0.05
β-myrcene	982		0.14	0.30	0.97	
α-terpinene	1011		0.17	0.34	0.68	
*p*-cymene	1014	1.65	9.33	15.61	23.33	1.54
limonene + 1,8 cineole	1023	0.31	0.57	0.93	1.43	1.38
*cis*-β-ocimene	1026		0.04	0.07		
γ-terpinene	1050	0.08	0.80	1.50	6.23	
*cis*-sabinene hydrate	1055	0.28	0.32	0.34		0.33
terpinolene	1081			0.06	0.08	
linalool	1084	0.46	0.83	0.56	1.89	0.50
camphor	1124	0.09	0.07		0.60	
borneol	1152	1.51	1.31	0.75	1.21	0.88
4-terpineol	1165	2.21	1.88	1.39	1.65	1.24
α-terpineol	1174	0.23	0.20	0.43	0.34	0.39
estragole	1176		0.32			
dihydrocarvone	1180	0.04	0.10			
thymol methyl ether	1215	0.27	0.14		4.33	0.17
carvacrol methyl ether	1226	0.64	1.14	1.12	1.98	0.38
cis-geraniol	1236				0.10	0.48
anethole	1261					
thymol	1267	4.42	7.60	10.11	40.87	3.06
carvacrol	1282	81.44	68.63	60.15	2.99	76.86
thymolacetate	1326				0.10	
α-bourbonene	1388		0.06	0.12	0.11	0.06
β-caryophyllene	1423	0.99	1.62	1.73	3.12	0.89
α-humulene	1456	0.10	0.17	0.16	0.10	0.08
γ-muurolene	1474	0.07	0.08	0.08	0.15	0.08
germacrene D	1481			0.07	0.07	
bicyclogermacrene	1496		0.06		0.08	
β-bisabolene	1503	0.41	0.60	0.68	0.07	0.24
γ-cadinene	1511	0.06	0.07	0.07	0.40	0.09
calamenene	1514	0.10	0.18	0.12	0.14	0.18
δ-cadinene	1518	0.12	0.13	0.14	0.33	0.16
spathulenol	1569	0.14	0.09	0.11		0.22
caryophyllene oxide	1576	1.23	0.81	0.82	1.43	1.86
Total		97.73	98.82	99.34	97.14	91.55

^1^ Retention indexes relative to standard mixture of n-alkanes on DB1-MS column.

**Table 5:** Chemical composition of oregano EOs OV07–OV11. Data are expressed as relative GC–MS% abundance of all detected components.

EO Component	RI ^1^	OV07	OV08	OV09	OV10	OV11
α-thujene	925	0.24	0.96			1.60
α-pinene	933	0.20	0.58	0.09	0.05	0.91
β-thujene	937		0.06			0.11
camphene	947	0.07	0.26			0.36
1-octen-3-ol	961	0.76	0.59	0.93	0.45	0.68
3-octanone	964	0.16	0.19	0.22	0.17	0.20
sabinene	968	0.08	0.27			0.08
β-pinene	974		0.19			0.26
3-octanol	978			0.05		
β-myrcene	982	0.29	0.64	0.06		0.98
α-terpinene	1011	0.36	0.97	0.09	0.06	0.90
*p*-cymene	1014	11.37	21.86	4.42	4.18	36.53
limonene + 1,8 cineole	1023	0.46	0.72	0.44	0.53	0.99
*cis*-*β*-ocimene	1026	0.05	0.12			0.18
γ-terpinene	1050	1.32	2.74	0.26	0.10	2.53
*cis*-sabinene hydrate	1055	0.76	0.45	0.33	0.73	0.13
terpinolene	1081	0.07	0.15			0.13
linalool	1084	0.65	0.52	0.58	1.01	0.25
camphor	1124	0.16	0.14	0.09	0.19	
borneol	1152	0.77	0.92	1.03	1.30	0.57
4-terpineol	1165	2.75	1.99	2.38	3.46	1.40
α-terpineol	1174	0.49	0.38	0.23	0.36	0.15
estragole	1176			0.29	0.71	
dihydrocarvone	1180			0.12	0.23	
thymol methyl ether	1215	0.40	0.13	0.19	0.41	0.10
carvacrol methyl ether	1226	1.14	1.11	0.90	1.24	1.25
cis-geraniol	1236					
anethole	1261				0.29	
thymol	1267	11.60	3.99	4.29	10.60	1.86
carvacrol	1282	58.76	54.91	78.33	65.58	44.72
thymolacetate	1326					
α-bourbonene	1388	0.07	0.07	0.05	0.07	0.06
β-caryophyllene	1423	2.24	2.24	0.95	1.18	1.20
α-humulene	1456	0.20	0.18	0.11	0.11	0.11
γ-muurolene	1474	0.10	0.07	0.06	0.08	
germacrene D	1481		0.17			
bicyclogermacrene	1496	0.14				
β-bisabolene	1503	0.54	0.53	0.38	0.48	0.39
γ-cadinene	1511	0.12	0.10	0.07	0.15	
calamenene	1514	0.12	0.27	0.10	0.31	
δ-cadinene	1518	0.21	0.16	0.12	0.21	0.08
spathulenol	1569	0.34	0.10	0.13	0.17	
caryophyllene oxide	1576	1.42	0.58	1.19	2.81	0.60
Total		98.39	99.28	98.46	97.20	99.28

^1^ Retention indexes relative to standard mixture of n-alkanes on DB1-MS column.

**Table 6:** Chemical composition of thyme EOs TV03–TV07. Data are expressed as relative GC–MS% abundance of all detected components.

EO Component	RI ^1^	TV03	TV04	TV05	TV06	TV07
methyl-2-methyl butanoate	757			0.13	0.04	0.14
α-thujene	925			0.94		0.85
α-pinene	933	0.11		0.61	0.06	0.69
camphene	947	0.10		0.37	0.05	0.35
1,4-pentenylpropionate	956	0.05		0.12	0.08	
1-octen-3-ol	961	1.00	0.93	0.94	1.23	0.59
3-octanone	964	0.12	0.08	0.09	0.08	0.06
β-pinene	974	0.06		0.17		0.22
3-octanol	978	0.13	0.10	0.11	0.15	0.06
β-myrcene	982	0.22		1.30	0.11	1.11
α-phellandrene	1000			0.18		
3-carene	1008			0.15		0.10
α-terpinene	1011	0.18		0.69	0.13	0.44
*p*-cymene	1014	9.61	3.94	25.44	4.63	32.24
1.8-cineole	1023	1.45	0.84	1.90	1.13	1.67
γ-terpinene	1050	2.72		7.63	2.02	1.61
cis-sabinene-hydrate	1055				0.12	
fenchone	1071			0.34		
linalool	1084	4.05	3.62	2.90	3.90	1.57
camphor	1124	0.80	0.76	0.87	0.50	
borneol	1152	2.28	1.77	1.34	1.87	0.76
4-terpineol	1165	2.47	2.36	1.97	2.36	1.40
α-terpineol	1174	0.59		0.31		0.35
estragole	1176		0.56	0.45	0.48	
thymol methyl ether	1215	1.76	1.03	1.50	1.10	1.58
carvacrol methyl ether	1225	0.90	0.50	0.86	0.58	0.86
*cis*-geraniol	1238	0.11	0.13	0.07	0.13	0.09
geranial	1246	0.20	0.13	0.10	0.10	
anethole	1262			0.17		
thymol	1267	55.20	64.73	38.40	61.18	35.66
carvacrol	1282	5.30	9.59	2.98	5.17	9.24
thymolacetate	1327			0.09	0.12	
α-copaene	1380					0.08
β-bourbonene	1388	0.10		0.09	0.08	0.11
β-caryophyllene	1423	1.99	0.94	2.43	2.44	1.95
β-farnesene	1448				0.07	
α-humulene	1456	0.07		0.08	0.08	0.08
γ-muurolene	1474	0.14	0.14	0.13	0.14	0.25
bicyclogermacrene	1496	0.07	0.09	0.07	0.08	0.13
β-bisabolene	1503	0.10		0.15	0.06	0.11
γ-cadinene	1511	0.32	0.27	0.36	0.29	0.34
calamenene	1514	0.13	0.27	0.11	0.12	0.21
δ-cadinene	1518	0.31	0.35	0.27	0.34	0.51
caryophyllene oxide	1576	2.88	2.40	1.68	1.77	1.67
Total		95.52	95.53	98.49	92.79	97.08

^1^ Retention indexes relative to standard mixture of n-alkanes on DB1-MS column.

**Table 7:** Chemical composition of thyme EOs TV08–TV11. Data are expressed as relative GC–MS% abundance of all detected components.

EO Component	RI ^1^	TV08	TV09	TV10	TV11
methyl-2-methyl butanoate	757	0.06	0.07		0.05
α-thujene	925	0.04	0.25		0.10
α-pinene	933	0.12	0.49	0.04	0.20
camphene	947	0.09	0.14		0.14
1,4-pentenylpropionate	956	0.05			0.06
1-octen-3-ol	961	1.47	0.82	0.77	0.70
3-octanone	964	0.12	0.09	0.06	0.08
β-pinene	974	0.05	0.09		0.07
3-octanol	978	0.21	0.08	0.13	0.10
β-myrcene	982	0.10	0.61		0.42
α-phellandrene	1000		0.06		
3-carene	1008				
α-terpinene	1011	0.08	0.31		0.35
*p*-cymene	1014	5.25	19.05	1.77	19.30
1.8-cineole	1023	1.92	1.89	0.81	1.09
γ-terpinene	1050	0.89	0.48	0.28	0.84
cis-sabinene-hydrate	1055			0.25	
fenchone	1071			0.21	
linalool	1084	3.36	3.02	3.40	2.65
camphor	1124	1.14	0.77	0.92	0.63
borneol	1152	1.60	1.08	1.91	1.34
4-terpineol	1165	2.58	2.22	1.94	1.70
α-terpineol	1174		0.25	0.19	
estragole	1176	0.57	1.23	0.91	0.72
thymol methyl ether	1215	0.23	2.40	1.07	1.54
carvacrol methyl ether	1225	0.28	0.95	0.79	0.96
*cis*-geraniol	1238	0.22	0.09	0.19	0.14
geranial	1246		0.07		
anethole	1262		0.78	1.48	0.28
thymol	1267	59.97	45.01	64.30	50.62
carvacrol	1282		7.68	6.63	5.02
thymolacetate	1327	0.06		0.10	0.06
α-copaene	1380	0.05	0.12		0.09
β-bourbonene	1388	0.11	0.15	0.06	0.11
β-caryophyllene	1423	3.04	3.91	1.62	3.01
β-farnesene	1448	0.17			
α-humulene	1456	0.11	0.14	0.07	0.12
γ-muurolene	1474	0.26	0.39	0.18	0.28
bicyclogermacrene	1496	0.11	0.18	0.10	0.17
β-bisabolene	1503	0.06	0.26	0.06	0.14
γ-cadinene	1511	0.29	0.62	0.27	0.36
calamenene	1514	0.19	0.37	0.20	0.28
δ-cadinene	1518	0.48	0.68	0.44	0.61
caryophyllene oxide	1576	3.10	1.58	2.14	1.21
Total		88.43	98.38	93.29	95.54

^1^ Retention indexes relative to standard mixture of n-alkanes on DB1-MS column.

**Table 8:** MIC determined on FV EO samples against the *S. aureus* and *P. aeruginosa* strains. Data are reported as % *v*/*v*.

EOs	6538P	25923	PA01	PA14
FV01	>5	>5	>5	>5
FV02	>5	>5	>5	>5
FV03	>5	>5	>5	>5
FV04	>5	>5	>5	>5
FV05	>5	>5	>5	>5
FV06	>5	>5	>5	>5
FV07	>5	>5	>5	>5
FV08	>5	>5	>5	>5
FV09	>5	>5	>5	>5
FV10	>5	>5	>5	>5
FV11	2.5	2.5	>5	>5
FV12	>5	>5	>5	>5
FV13	>5	>5	>5	>5

**Table 9:** MIC determined on OV EO samples against the *S. aureus* and *P. aeruginosa* strains. Data are reported as % *v*/*v*.

EO Name	6538P	25923	PA01	PA14
OV01	NT	NT	NT	NT
OV02	NT	NT	NT	NT
OV03	0.31	0.31	>5	>5
OV04	0.16	1.25	>5	>5
OV05	2.5	>5	>5	>5
OV06	NT	NT	NT	NT
OV07	0.16	0.31	>5	>5
OV08	0.16	1.25	>5	>5
OV09	1.25	0.62	>5	>5
OV10	NT	NT	NT	NT
OV11	2.5	2.5	>5	>5

NT: Not Tested.

**Table 10:** MIC determined on TV EO samples against *S. aureus* and *P. aeruginosa* strains. Data are reported as % *v*/*v*.

EO Name	6538P	25923	PA01	PA14
TV01	NT	NT	NT	NT
TV02	NT	NT	NT	NT
TV03	NT	NT	NT	NT
TV04	NT	NT	NT	NT
TV05	NT	NT	NT	NT
TV06	0.16	0.31	>5	>5
TV07	0.31	0.31	>5	>5
TV08	0.31	0.31	>5	>5
TV09	NT	NT	NT	NT
TV10	NT	NT	NT	NT
TV11	0.31	0.16	>5	>5

NT: Not Tested.

**Table 11:** Details on the soil treatment for the growth of the three plants investigated in this study.

Treatment	Description
Control	Absence of fertilization; the plant growth does not depend on the nitrogen supplied but rather the amount of phosphorus and potassium found in the untreated soil.
Mineral	Addition of a chemical fertilizer which releases to the soil 11 kg/hectare of nitrogen, 12 kg/hectare of phosphorus and 16 kg/hectare of potassium.
Organic–Mineral	Treatment with Berfoss Bio 3-11, a fertilizer with high agronomic yield, with hydrolyzed organic substance at acid pH for the maintenance and enrichment of the available phosphorus endowment; this supplies the soil with 3 kg/hectare of nitrogen and 11 kg/hectare of phosphorus.
Organic	Bioilsa Basic; natural-origin organic and organo-mineral fertilizers with a high content of organic nitrogen of protein origin with modulated release that release to the soil 2 kg/hectare of nitrogen.

## Data Availability

Not applicable.

## Supplementary Materials

The following supporting information can be downloaded at: https://www.mdpi.com/article/10.3390/plants12152835/s1, Table S1: List of quantified constituents of FV essential oils. Retention indexes and method of identification used.; Table S2: List of quantified constituents of OV essential oils. Retention indexes and method of identification used; Table S3: List of quantified constituents of TV essential oils. Retention indexes and method of identification used.
