# Supplementary material for: Effect of Different Soil Treatments on Production and Chemical Composition of Essential Oils Extracted from Foeniculum vulgare Mill., Origanum vulgare L. and Thymus vulgaris L
Source: Plants (Basel). 2023 Jul 31;12(15):2835. doi: 10.3390/plants12152835 (PMC10421082; doi:10.3390/plants12152835)
Supplement: Supplementary file 1 [file plants-12-02835-s001.zip › plants-2490222-supplementary.pdf]

Supplementary Material

Effect of Different Soil Treatments on Production and Chemical Composition of Essential Oils Extracted from *Foeniculum vulgare* Mill., *Origanum vulgare* L. and *Thymus vulgaris* L.

Antonio Raffo [1,¥], Filippo Umberto Sapienza [2,¥], Roberta Astolfi [2], Gabriele Lombardi [3], Caterina Fraschetti [4], Mijat Božović [5], Marco Artini [6], Rosanna Papa [6], Marika Trecca [6], Simona Fiorentino [7], Valerio Vecchiarelli [7], Claudia Papalini [8], Laura Selan [6] and Rino Ragno [2,*]

[1] CREA-Research Centre for Food and Nutrition, Via Ardeatina, 546, 00178 Rome, Italy; antonio.raffo@crea.gov.it
[2] Rome Center for Molecular Design, Department of Drug Chemistry and Technology, Sapienza University, p.le Aldo Moro 5, 00185 Rome, Italy; filippo.sapienza@uniroma1.it; astolfi.1766291@studenti.uniroma1.it
[3] Department of Environmental Biology, Sapienza University, p.le Aldo Moro 5, 00185 Rome, Italy; gabriele.lombardi@uniroma1.it
[4] Department of Drug Chemistry and Technology, Sapienza University, p.le Aldo Moro 5, 00185 Rome, Italy; caterina.fraschetti@uniroma1.it
[5] Faculty of Natural Science and Mathematics, University of Montenegro, Džordža Vašingtona bb, 81000 Podgorica, Montenegro; mijatboz@ucg.ac.me
[6] Department of Public Health and Infectious Diseases, Sapienza University, p.le Aldo Moro 5, 00185 Rome, Italy; marco.artini@uniroma1.it; rosanna.papa@uniroma1.it; trecca.1820515@studenti.uniroma1.it; laura.selan@uniroma1.it
[7] Centro Appenninico del Terminillo "Carlo Jucci". Perugia University. Via Comunali,43 – 02100 Rieti, Italy; simona.fiorentino@unipg.it; valerio.vecchiarelli@unipg.it
[8] ARSIAL Agenzia Regionale per lo Sviluppo e l'Innovazione dell'Agricoltura del Lazio, Rome, Italy; c.papalini@arsial.it
[*] Correspondence: rino.ragno@uniroma1.it; Tel.: +39-6-4991-3937; fax: +39-6-4991-3627.
[¥] These authors contributed equally to this work.

Table S1. List of quantified constituents of FV essential oils. Retention indexes and method of identification used.

Nr	Compound name	Retention index experimental	Retention index, literature[1]	Method of identification[2]
1	α -pinene	933	924-951	RI, MS, PC
2	sabinene	968	958-981	RI, MS
3	β-pinene	974	962-987	RI, MS, PC
4	β-myrcene	982	975-991	RI, MS, PC
5	α-phellandrene	1000	990-1009	RI, MS, PC
6	3-carene	1008	997-1027	RI, MS
7	*p*-cymene	1014	1004-1029	RI, MS, PC
8	limonene	1023	1012-1038	RI, MS, PC
9	γ-terpinene	1050	1035-1062	RI, MS, PC
10	fenchone	1071	1059-1087	RI, MS
11	linalool	1084	1074-1098	RI, MS, PC
12	fenchyl alcohol	1105	1088-1122	RI, MS
13	cis-*p*-menth-2,8-dienol	1118	1100-1127	RI, MS
14	camphor	1124	1106-1153	RI, MS, PC
15	4-terpineol	1165	1148-1180	RI, MS, PC

16	estragole	1176	1169-1190	RI, MS, PC
17	verbenone	1185	1167-1198	RI, MS, PC
18	fenchylacetate, endo	1209	1205-1215	RI, MS
19	p-anisaldehyde	1215	1206-1240	RI, MS
20	fenchylacetate, exo	1224	1214-1232	RI, MS
21	anethole	1261	1253-1284	RI, MS, PC
22	isobornyl acetate	1272	1259-1282	RI, MS
23	carvacrol	1282	1272-1300	RI, MS, PC
24	2,3-dimethylhydroquinone	1333	n.a.	MS
25	anisyl methyl ketone	1343	n.a.	MS
26	β-caryophyllene	1423	1400-1442	RI, MS, PC
27	4-methoxycinnamaldehyde	1520	1505-1536	RI, MS
28	caryophyllene oxide	1576	1549-1587	RI, MS

Notes: [1] Reported range are the 90% confidence interval estimates of retention indexes, based on the number of available data records, determined on dimethylsilicone stationary phase, as reported by Babushok et al., 2011.
[2] Identification of compound based on: RI (literature retention index), MS (library mass spectrum), PS (retention index and mass spectrum of pure standard compound).

Table S2. List of quantified constituents of OV essential oils. Retention indexes and method of identification used.

Nr	Compound name	Retention index experimental	Retention index, literature[1]	Method of identification[2]
1	α-thujene	925	916-938	RI, MS
2	α -pinene	933	924-951	RI, MS, PC
3	β-thujene	937	n.a.	MS
4	camphene	947	936-965	RI, MS
5	1-octen-3-ol	961	958-980	RI, MS, PC
6	3-octanone	964	961-971	RI, MS, PC
7	sabinene	968	958-981	RI, MS
8	β-pinene	974	962-987	RI, MS, PC
9	3-octanol	978	974-995	RI, MS
10	β-myrcene	982	975-991	RI, MS, PC
11	α-terpinene	1011	1001-1024	RI, MS, PC
12	*p*-cymene	1014	1004-1029	RI, MS, PC
13	limonene<br>+ 1,8-cineole	1023	1012-1038<br>1013-1039	RI, MS, PC<br>RI, MS, PC
14	*cis*-β-ocimene	1026	1017-1040	RI, MS
15	γ-terpinene	1050	1035-1062	RI, MS, PC
16	*cis*-sabinene hydrate	1055	1044-1066	RI, MS
17	terpinolene	1081	1064-1091	RI, MS, PC
18	linalool	1084	1074-1098	RI, MS, PC
19	camphor	1124	1106-1153	RI, MS, PC
20	borneol	1152	1134-1172	RI, MS, PC
21	4-terpineol	1165	1148-1180	RI, MS, PC
22	estragole	1176	1169-1190	RI, MS, PC
23	dihydrocarvone	1180	1162-1206	RI, MS
24	thymol methyl ether	1215	1199-1235	RI, MS
25	carvacrol methyl ether	1225	1205-1230	RI, MS
26	cis-geraniol	1236	1231-1256	RI, MS
27	anethole	1261	1253-1284	RI, MS
28	thymol	1267	1260-1289	RI, MS, PC
29	carvacrol	1282	1272-1300	RI, MS, PC
30	thymol acetate	1326	1330-1351	RI, MS
31	α-bourbonene	1388	1383-1409	RI, MS
32	β-caryophyllene	1423	1400-1442	RI, MS, PC
33	α-humulene	1456	1430-1466	RI, MS, PC
34	γ-muurolene	1474	1455-1494	RI, MS
35	germacrene D	1481	1458-1491	RI, MS
36	bicyclogermacrene	1496	1474-1501	RI, MS
37	β-bisabolene	1503	1485-1511	RI, MS
38	γ-cadinene	1511	1490-1521	RI, MS
39	calamenene	1514	1505-1524	RI, MS
40	δ-cadinene	1518	1498-1526	RI, MS

41	spathulenol	1569	1549-1580	RI, MS
42	caryophyllene oxide	1576	1549-1587	RI, MS

Notes: [1] Reported range are the 90% confidence interval estimates of retention indexes, based on the number of available data records, determined on dimethylsilicone stationary phase, as reported by Babushok et al., 2011.

[2] Identification of compound based on: RI (literature retention index), MS (library mass spectrum), PS (retention index and mass spectrum of pure standard compound).

Table S3. List of quantified constituents of TV essential oils. Retention indexes and method of identification used.

Nr	Compound name	Retention index experimental	Retention index, literature[1]	Method of identification[2]
1	methyl-2-methyl butanoate	757	755-768	RI, MS
2	α-thujene	925	916-938	RI, MS
3	α -pinene	933	924-951	RI, MS, PC
4	camphene	947	936-965	RI, MS
5	1,4-pentenylpropionate	956	956-990	RI, MS
6	1-octen-3-ol	961	958-980	RI, MS, PC
7	3-octanone	964	961-971	RI, MS, PC
8	β-pinene	974	962-987	RI, MS, PC
9	3-octanol	978	974-995	RI, MS
10	β-myrcene	982	975-991	RI, MS, PC
11	α-phellandrene	1000	990-1009	RI, MS, PC
12	3-carene	1008	997-1027	RI, MS
13	α-terpinene	1011	1001-1024	RI, MS, PC
14	*p*-cymene	1014	1004-1029	RI, MS, PC
15	1,8-cineole	1023	1013-1039	RI, MS, PC
16	γ-terpinene	1050	1035-1062	RI, MS, PC
17	*cis*-sabinene hydrate	1055	1044-1066	RI, MS
18	fenchone	1071	1059-1087	RI, MS
19	linalool	1084	1074-1098	RI, MS, PC
20	camphor	1124	1106-1153	RI, MS, PC
21	borneol	1152	1134-1172	RI, MS, PC
22	4-terpineol	1165	1148-1180	RI, MS, PC
23	α-terpineol	1174	1159-1191	RI, MS, PC
24	estragole	1176	1169-1190	RI, MS, PC
25	thymol methyl ether	1215	1199-1235	RI, MS
26	carvacrol methyl ether	1225	1205-1230	RI, MS
27	cis-geraniol	1236	1231-1256	RI, MS
28	geranial	1246	1236-1260	RI, MS
29	anethole	1261	1253-1284	RI, MS
30	thymol	1267	1260-1289	RI, MS, PC
31	carvacrol	1282	1272-1300	RI, MS, PC
32	thymol acetate	1326	1330-1351	RI, MS
33	α-copaene	1380	1360-1392	RI, MS
34	β-bourbonene	1388	1383-1409	RI, MS
35	β-caryophyllene	1423	1400-1442	RI, MS, PC
36	β-farnesene	1448	1438-1466	RI, MS
37	α-humulene	1456	1430-1466	RI, MS, PC
38	γ-muurolene	1474	1455-1494	RI, MS
39	bicyclogermacrene	1496	1474-1501	RI, MS

40	β-bisabolene	1503	1485-1511	RI, MS
41	γ-cadinene	1511	1490-1521	RI, MS
42	calamenene	1514	1505-1524	RI, MS
43	δ-cadinene	1518	1498-1526	RI, MS
44	caryophyllene oxide	1576	1549-1587	RI, MS

Notes: [1] Reported range are the 90% confidence interval estimates of retention indexes, based on the number of available data records, determined on dimethylsilicone stationary phase, as reported by Babushok et al., 2011.
[2] Identification of compound based on: RI (literature retention index), MS (library mass spectrum), PS (retention index and mass spectrum of pure standard compound).
